# Supplementary material for: Lower Critical Solution Temperature Tuning and Swelling Behaviours of NVCL-Based Hydrogels for Potential 4D Printing Applications
Source: Polymers (Basel). 2022 Aug 2;14(15):3155. doi: 10.3390/polym14153155 (PMC9370960; doi:10.3390/polym14153155)
Supplement: Supplementary file 1 [file polymers-14-03155-s001.zip › polymers-1799212-supplementary.pdf]

Supplementary Figures

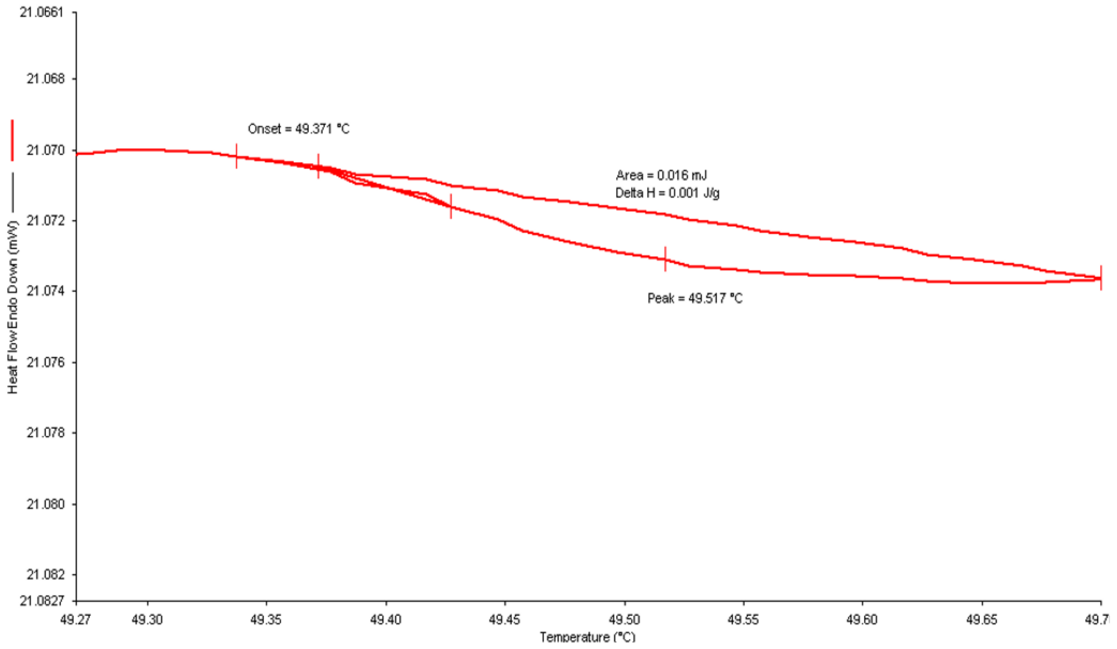

Figure S1. The thermogram illustrating the LCST of PNVCL based copolymer S1 (30NVP/70NVCL) aqueous solution.

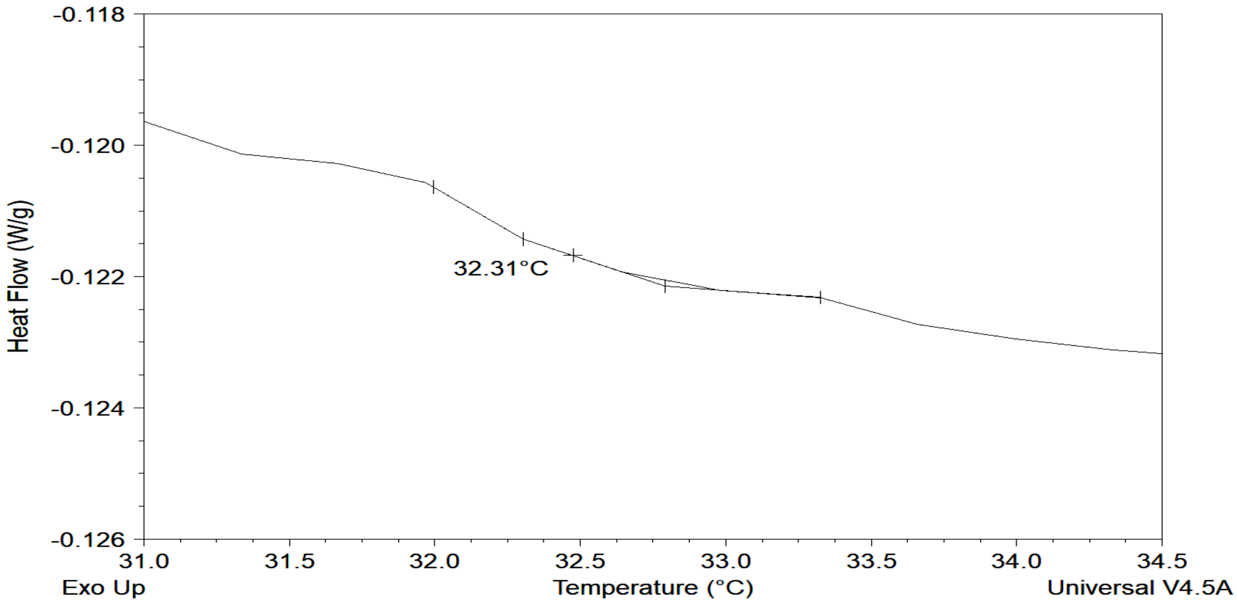

Figure S2. The thermogram illustrating the LCST of NVCL based copolymer S2 (30DMAAm/70NVCL) aqueous solution.

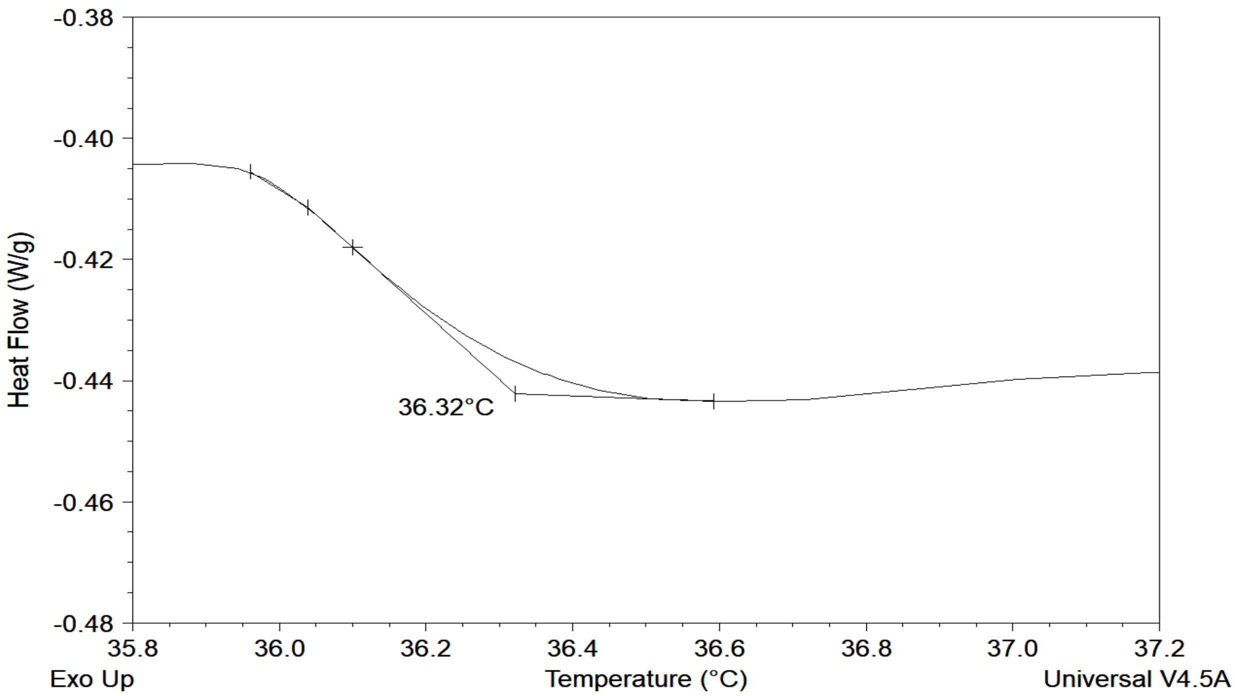

Figure S3. The thermogram illustrating the LCST of NVCL based terpolymer S3 (15NVP/15DMAAm/70NVCL) aqueous solution.
